# Owner assessed outcomes following elbow arthroscopy with or without platelet rich plasma for fragmented medial coronoid process

**DOI:** 10.3389/fvets.2022.938706

**Published:** 2022-08-02

**Authors:** Alyssa M. Matos Cruz, David R. Mason

**Affiliations:** ^1^MedVet Columbus, Worthington, OH, United States; ^2^Las Vegas Veterinary Specialty Center, Las Vegas, NV, United States

**Keywords:** platelet rich plasma (PRP), elbow dysplasia, fragmented medial coronoid, arthroscopy, orthopedics, regenerative medicine

## Abstract

**Objective:**

Document the outcomes of bilateral arthroscopic subtotal coronoidectomy for the fragmented medial coronoid process, quantify persistent lameness that required additional treatment (PRP), and document the outcomes of dogs that followed up with PRP injections.

**Study design:**

Retrospective study.

**Sample population:**

Overall, 115 dogs underwent arthroscopy alone and 31 received PRP at least 6 weeks after arthroscopy. The owner's response rate was ~50% (73 dogs).

**Methods:**

Collected data included signalment, unilateral or bilateral clinical signs, intra-articular chondroprotective injection during the procedure, if PRP intra-articular injection was received postoperatively, and if it was received, the time from the initial surgery to administration was recorded. Outcomes were assessed *via* standardized owner questionnaires using the Liverpool Osteoarthritis in Dogs (LOAD) score, the Canine Brief Pain Inventory (CBPI) score, and the overall quality of life (QOL) assessment.

**Results:**

Approximately 20% of the patients received PRP post-operatively due to persistent lameness following surgery. Similar pain scores were found between the two groups with an average of 11–13 LOAD score, 13–15 CBPI score, and good quality of life. Older animals at the time of surgery and those that received pain-relieving medications after the procedure were more painful and affected their functional outcome. PRP as an adjunctive therapy achieved a perceived good to excellent quality of life in ~90% of pets in this population.

**Conclusion:**

Arthroscopy and subtotal coronoidectomy followed by PRP, if needed, seemed to decrease pain, and improve lameness in the long term.

**Clinical significance:**

PRP should be considered as adjunctive therapy in dogs with the limited response to arthroscopy alone.

## Introduction

Elbow dysplasia affects many large breed dogs worldwide and is considered an extremely debilitating disease due to its natural progression. The most common etiology of elbow arthrosis is the fragmented medial coronoid process (FMCP), after the cartilaginous process ossifies and the deeper layer of chondrocytes undergo malacia (1). One of the surgical treatments involve arthroscopic fragment removal and osteochondral debridement. However, this does not always eliminate the clinical signs related to pain and lameness, especially, in the older dogs (1). This is partly because cartilage has a poor-regenerative capacity. Platelet-rich plasma (PRP) is being used in human and veterinary medicine for many disease processes including joint disease, due to its growth factors and anti-inflammatory properties (2, 3). Intra-articular use of platelet-rich plasma has been a topic of investigation and debate due to its anabolic and anti-catabolic effects aiming to increase cartilage regenerative capacity and reduction of inflammatory mediators (2, 3). Fortier et al. reviewed *in vitro* laboratory investigations, preclinical animal model studies, and human clinical studies which collectively support the use of PRP for cartilage injuries and joint pain (4).

Platelet-rich plasma injections have been used in human medicine for osteoarthritis and treatment of inflammatory orthopedic conditions without a consensus of their efficacy (5–10). A meta-analysis of human studies did not find PRP intraarticular injections an effective treatment for osteoarthritis compared to hyaluronic acid (5). However, in another set of meta-analyses of the randomized controlled trials including over one thousand participants, intra-articular injections of PRP were reported to decrease pain and improve function levels up to 12 months when compared to placebo, hyaluronic acid, and corticosteroid injections (6–10).

In veterinary medicine, platelet-rich plasma has been used and reported as a treatment option for a variety of disease processes with favorable outcomes and no major complications (11–19). Platelet-rich plasma has been described as a treatment for supraspinatus tendinopathy (11), early partial cranial cruciate ligament tears (12), bilateral hip osteoarthritis (13), large cutaneous wounds (14), aural hematomas (15), lumbosacral stenosis (16), stifle osteoarthritis secondary to medically managed chronic cranial cruciate ligament tears (17), and for bone healing in traumatic fractures (18) and also high-tibial osteotomy (19). In these cases, all had favorable outcomes, except for use in high-tibial osteotomies in which there was no significant difference between control and experimental groups. Considering its low-complication risk and ease of collection and administration, PRP can have potential value as adjunctive therapy for cartilage defects and promoter of healing in medial compartment disease in dogs. To the author's knowledge, there have been no studies on the use of PRP for medial compartment disease after subtotal coronoidectomy and osteochondral debridement. Assessing the potential clinical benefits of PRP as adjunctive therapy for FMCP is paramount to improve the quality of life of pets and owner satisfaction after elbow arthroscopy in pets with this debilitating condition.

Our objectives were to document the outcome of bilateral arthroscopic subtotal coronoidectomy for the bilateral fragmented medial coronoid process and to quantify persistent lameness that required additional treatment. The second objective was to document the outcomes of the dogs that received platelet-rich plasma injection when refractory to surgical treatment alone. We hypothesized that overall dogs would have a good quality of life, good function, and low-pain levels with surgery alone or with adjunctive platelet-rich plasma when surgery alone did not achieve the desired clinical outcome.

## Materials and methods

### Inclusion criteria

The database of the Las Vegas Veterinary Specialty Center was reviewed for dogs that underwent elbow arthroscopy between 1 March 2013 and 30 March 2021, performed by the same board-certified surgeon (DM). Only dogs diagnosed with a bilateral fragmented medial coronoid process *via* arthroscopy that underwent a subtotal coronoidectomy were evaluated in this study. Dogs were excluded if they underwent arthroscopic surgery twice, received PRP during the procedure, or had other elbow or shoulder joint-related comorbidities (osteochondrosis dissecans, ununited anconeal process, and radio-ulnar incongruency). A minimum of 6 weeks past the surgery date was required to be included in the study.

### Data collection

Collected data included breed, weight, sex, age at the time of surgery, unilateral or bilateral clinical signs, intra-articular chondroprotective injection during the procedure (hyaluronic acid, HA, and polyglutamic acid, PGA), and if platelet-rich plasma intra-articular (IA) injection was received postoperatively, and the time from the initial surgery to the administration of platelet-rich plasma injection. The PRP injections were offered to clients that were still showing lameness at least 6 weeks after the procedure or lack of improvement as perceived by the owners.

### Outcome assessment

The outcomes were assessed *via* standardized owner questionnaires using the Liverpool Osteoarthritis in Dogs (LOADs) score, the Canine Brief Pain Inventory (CBPI) score, and the overall quality of life (QOL) assessment. The medications the pets were receiving at the time of the questionnaire were also recorded. Medications were categorized as an anti-inflammatory (prednisone and non-steroidal anti-inflammatory, NSAID), joint supplements, and miscellaneous pain medications (gabapentin, amantadine, and tramadol).

### Statistical methods

The response variables were the use of PRP (binary, 01), and the outcome of LOAD (continuous), CBPI (continuous), and QOL (ordinal). The factors that were described and tested for association with PRP were age, weight, number of sides clinically affected (unilateral vs. bilateral), and gender. Descriptive data were reported by the means of mean, SD, median, and 25th and 75th quartiles. Analysis was by multivariate logistic regression. The factors that were tested for association with the outcomes were PRP, gender, age, number of sides clinically affected, IA injection, and use of NSAID, supplements, or other pain medications. Analysis was by multiple linear regression. Multicollinearity was tested by means of variance inflation factor; <2.5 was considered acceptable. Linearity was tested by the means of residual plots. The *P*-values were reported.

## Results

There were 182 dogs diagnosed with a bilateral fragmented medial coronoid process *via* arthroscopy that underwent a subtotal coronoidectomy. In total, thirty-six dogs were excluded from the data analysis: two underwent elbow arthroscopy twice, two passed away from unrelated causes prior to completing the recovery period, five had unilateral FMCP, eight received PRP during the procedure, and nineteen with elbow or shoulder joint-related comorbidities (osteochondrosis dissecans, ununited anconeal process, and radio-ulnar incongruency).

Of the 146 dogs that met the inclusion criteria, 115 underwent surgery alone (SXA group). In total, thirty-one patients received PRP (PRP group) based on the physical examination and owner assessment of surgery success past the recovery period. In total, 73 owners answered the questionnaires (50% response rate). From the SXA group, 53 answered the questionnaire (46%). From the PRP group, 20 answered the questionnaire (65%). The questionnaires were answered following a minimum of 6 weeks up to 5 years postoperatively, which is the time PRP treatment became available to our population. The mean follow-up time for SAX was 1,220 days, for PRP was 1,269 days, with a total mean follow-up time of 1,234 days (about 3 and a half years) from the initial arthroscopic procedure day to the date the questionnaire was received.

The mean and median weight was 34 kg, mean age was 26 months (SD, 22) with a median of 18 months (about 1 and a half years), and the mean number of days until PRP was administered was 247 days (SD 355) with a median of 106. Dog's ages ranged from 5 to 92 months (7.5 years old). There was a total of 61 females, 11 intact and 50 spayed, and 85 males, 22 intact and 63 neutered. Most dogs were presented with unilateral clinical signs (104) and the rest presented with bilateral lameness or pain (41). Most, 128 dogs, received an intraarticular injection with hyaluronic acid gel perioperatively. At the time of the survey, 10 dogs were receiving anti-inflammatories (non-steroidal anti-inflammatories or prednisone), 17 were receiving supplements (including glycosaminoglycans, chondroitin, fish oil, etc.), and 9 were receiving other types of pain medications (including gabapentin, amantadine, or/and tramadol).

The overall (both groups) median and mean LOAD and CBPI scores, and QOL obtained from the questionnaires are summarized in Table 1. When the SXA group was analyzed separately from the PRP group the data was the following: SXA median LOAD was 11 (4–19), CBPI 3 (0–13), and QOL good (3). The mean LOAD was 13 (SD 9), CBPI was 13 (SD 23), and QOL was good (3, SD 1) (Table 2). For the PRP group, the median LOAD was 11 (4–19), CBPI 3 (0–13), and QOL good (3). The mean LOAD was 13 (SD 9), CBPI was 13 (SD 23), and QOL was good (3, SD 1) (Table 3). A summary of results when the overall patient results were evaluated for a threshold of 20 for LOAD, 30 for CBPI and good or better quality of life is shown in Table 4, which was 81%, 85%, and 95% of patients, respectively.

**Table 1:** Summary of all the cases.

**All data** ***n =* 146**	**Kg**	**Age (months)**	**LOAD**	**CBPI**	**QOL**
Mean	34.7	26.1	13.2	14.9	3.2
Median	34.0	18.0	11.0	4.0	3.0
SD	12.3	22.6	8.9	23.1	1.0
25th Percentile	27.0	10.0	6.5	0.0	3.0
75th Percentile	41.0	36.0	18.5	17.0	4.0

**Table 2:** Summary for arthroscopy alone (SXA).

**PRP= 0**	**Kg**	**Age (months)_**	**LOAD**	**CBPI**	**QOL**
Mean	34.0	26.9	12.6	13.0	3.2
Median	33.0	18.0	11.0	3.0	3.0
SD	10.9	22.9	9.4	23.4	1.0
25th Percentile	27.0	11.0	4.0	0.0	3.0
75th Percentile	40.0	36.0	18.5	13.5	4.0

**Table 3:** Summary of patients who received PRP (PRP).

**PRP= 1**	**Kg**	**Age (months)**	**Days from surgery to PRP treatment**	**LOAD**	**CBPI**	**QOL**
Mean	37.5	23.4	246.7	14.8	19.8	3.0
Median	35.0	15.0	106.0	13.5	14.0	3.0
SD	16.4	21.6	355.7	7.4	22.0	0.8
25th Percentile	27.0	9.0	67.0	8.0	5.8	2.3
75th Percentile	45.0	33.0	168.0	21.0	20.8	3.8

**Table 4:** Overall outcomes summary.

	**QOL good, very good, excellent**	**LOAD <20**	**CBPI <30**
SXA (53)	50	44	46
PRP (20)	19	15	16
Total (73)	69	59	62
Total percentage	95%	81%	85%

The use of PRP on refractory cases was not associated with any factor (age, weight, if one or both elbows were clinical at the time of initial presentation, gender, or neuter status). LOAD scores were influenced by the use of anti-inflammatories and age. LOAD was 11 units higher for those using anti-inflammatories, and for each month increase in age, LOAD increased by 0.11. Similarly, CBPI was influenced by the use of pain medications and age. CBPI increased by 0.5 with each month of age and if miscellaneous pain medications were given, CBPI was 18.2 higher. The QOL decreased with age and if other pain medications were given (Table 5). Adjunctive treatment with PRP injections showed no statistical significance for outcomes of LOAD and QOL, but seemed to be significantly associated with CBPI, *P*-value of 0.03 (Table 6).

**Table 5:** Regression analysis summary.

	**LOAD**	**CBPI**	**QOL**
Age (months)	0.11	0.53	−0.02
NSAID	11.06	N/A*	N/A*
Miscellaneous pain medications	N/A*	18.20	−0.69

*N/A when P-value was >0.1 or variance inflation factor >2.5.

**Table 6:** Significance of PRP on the outcome (univariate analysis).

**Outcome**	***P*-value**
LOAD	0.20
CBPI	0.03
QOL	0.1

## Discussion

Based on our data collection, ~20% (31/146) of the owners whose pets underwent the arthroscopic procedures at our hospital were unhappy with surgery results (persistent lameness) and received PRP at least 6 weeks postoperatively but mostly after 3 months to a year postsurgery. The overall outcome of both groups when analyzed together and separately was favorable, with an average of 11–13 of a total of 52 LOAD score, and of 13–15 of a possible 100 total CBPI score, and good quality of life. No statistical difference in outcome was found between the two groups, except for CBPI. However, the significance is questionable given the strong selection bias for the patients receiving PRP.

As depicted in Table 4, a total of 95% of all the owners considered their dogs to have a good, very good, or excellent quality of life. Approximately 81% had a LOAD score <20 which is equivalent to mild-to-moderate osteoarthritic changes based on the LOAD guidelines, and 85% had a CBPI <30 which rates the severity of their dog's pain and the degree to which that pain interferes with function on a scale of 1–10 for 10 questions (maximum score of 100). With that in mind, ~87% of our population benefited from surgery and PRP injections when needed.

Our results also showed that older animals at the time of surgery seemed to have worse outcomes and those that were maintained on pain-relieving medications for long term were also considered to be more painful and with negative effects on their functional long-term outcome. This is most likely due to the progression of joint arthrosis and arthritis seen with the medial compartment disease. It is possible that when left untreated these changes might occur more rapidly, suggesting that surgery should be pursued early on if FMCP is suspected as reported previously (20–22). The anti-inflammatory use factor is not surprising as it is widely preferred as the first line of treatment for pain in the orthopedic disease.

Our 20% unsatisfaction rate after arthroscopic evaluation and subtotal coronoidectomy, was similar to that previously reported by Fitzpatrick et al. (20). In their study, ~45% of owners were satisfied with results at 5 weeks postoperatively, and 96% at 24 weeks (about 5 and a half months). In our study, the difference in lameness from 6 weeks postoperatively (initial recheck) vs. long term was not able to be accurately measured and evaluated because of the lack of complete clinical records or lack of in person rechecks. Owner phone updates were requested in many of our cases and only followed up with a minority if there was a lack of improvement. When long-term outcome (1–6 years postoperatively) was evaluated, the percentage of satisfaction and sound dogs was similar to our results at 80% (23).

Previous studies using owner-assessed outcomes after treatment of a fragmented medial coronoid process seemed to be in accordance with our results. In a small study of Bernese Mountain dogs, although there was decreased range of motion and increased radiographic evidence of osteoarthritis, all the owners reported improved lameness and function (24). In another prospective study, although only some of the dogs were completely sound, all the owners were satisfied with surgery outcomes at 6–24 months (about 2 years) postoperatively (25). When looking at the more objective measurements using kinematic analysis up to 180 days (about 6 months) post arthroscopy, a slight improvement was noted after surgery (26). It has been reported that function improves regardless of incongruency and cartilage erosion present, although the improvement was more significant in the latter, suggesting addressing incongruency might not be as beneficial long term which is beyond the scope of this study (27, 28).

Although arthroscopy and subtotal coronoidectomy generally is the treatment of choice for specialty care regarding medial compartment disease, there are multiple studies that did not find an appreciable long-term difference when compared with the medically managed patients at 12 months (1 year) and 56 months (4.6 years), respectively (29, 30). Moreover, recent studies have evaluated the elbow joint objectively and found that there are calcified bodies remaining or formed after arthroscopy. In one study looking at recurrent lameness with computed tomography (CT) or arthroscopic re-evaluation, it was reported that after a mean of 3 years progressive osteoarthritis and cartilage damage was seen in the form of loose scar tissue or calcified bodies (31). Of 29 dogs evaluated, 12 had calcified bodies, and 14 had scar tissue formation suspected secondary to the first arthroscopic procedure (31). In contrast, Renner et al. (32) evaluated immediate or short-term postoperative CT scans for bony remnants and found that 73% of cases had remaining lesions, mostly at the radial incisure. That study reported that only 5% of radial incisure lesions were removed and 72% of apical lesions were removed completely. Different surgeons, techniques, and different lesion locations could pose another variable for outcomes. Although the specific location of lesions was not specified, the technique and surgeon variability were controlled in this study. If there are secondary changes or loose fragments because of the surgical procedure as implied in the mentioned study, it might be worth considering anti-inflammatory and cartilage effects of PRP at the time of surgery instead of only in a selected number of cases.

Due to its retrospective nature and subjective measurements, this study has several limitations. The timeframe and follow-up period varied between the two groups, preventing direct comparisons. The PRP group lacks a baseline score or objective evaluation of joints to evaluate significant improvement before and after additional treatment, and the source of pain and recurrent lameness. There is also a financial burden with additional therapy that is not accounted for when evaluating and assuming success on those that did not follow-up or did not receive PRP. Although most owners were satisfied with arthroscopy regardless of PRP administration, the low-response rate (50%), prevents the ability to draw conclusions. Establishing accurate conclusions on surgical outcomes based on owner assessment of pain levels in dogs is difficult. Many subjective factors can influence owner satisfaction such as activity level, expense, and client–staff interactions. Objective data, such as force plate analysis and kinematic gait evaluations, and a prospective controlled study design could strengthen our conclusions.

Overall, our results showed long-term benefit to dogs with medial compartment disease due to FMCP with arthroscopic subtotal coronoidectomy, subchondral debridement, and PRP when used as an adjunctive therapy. Not all the dogs needed PRP for successful outcomes but it seems to help the 20% of the population where surgery alone is not as effective. When surgery was not successful according to owners, PRP seemed to improve the degree of lameness long term as dogs were improved at recheck and did not need further rounds of the PRP injections. Owners reported low pain levels as described by LOAD and CBPI scores, and good quality of life. To date, there are no clinical studies looking at PRP for elbow joint pain or disease. Approximately 81–95% of our population post surgery with or without PRP injections had a good function and low-pain levels.

This percentage might be different given the limitation of lack of owner response and recheck timeframe. For the minority of cases that received PRP after 6 weeks prior to a full recovery (~3–4 months), an argument can be made that they might have had a full recovery without the need for PRP treatment. At the time of the study, further rounds of PRP were being offered to those patients that remained lame. It would have been helpful to ask the owners if they would have or would pursue PRP based on their pet's lameness as some owners might have several reasons unrelated to the clinical signs for not following up with additional therapy, including financial, lifestyle, or other personal reasons.

Platelet-rich plasma therapy should be considered as adjunctive therapy for those non-responsive or with a limited response to arthroscopy because of their growth factors and anti-inflammatory properties. However, further investigation on PRP therapy in dogs, and its effects on cartilage repair are necessary to make definitive treatment plans in the future.

## Data availability statement

The original contributions presented in the study are included in the article/supplementary material, further inquiries can be directed to the corresponding author.

## Author contributions

All authors listed have made a substantial, direct, and intellectual contribution to the work and approved it for publication.

## Conflict of interest

The authors declare that the research was conducted in the absence of any commercial or financial relationships that could be construed as a potential conflict of interest.

## Publisher's note

All claims expressed in this article are solely those of the authors and do not necessarily represent those of their affiliated organizations, or those of the publisher, the editors and the reviewers. Any product that may be evaluated in this article, or claim that may be made by its manufacturer, is not guaranteed or endorsed by the publisher.
